# The Selection and Validation of Reference Genes for RT-qPCR Analysis of the Predatory Natural Enemy *Orius nagaii* (Hemiptera: Anthocoridae)

**DOI:** 10.3390/insects15120936

**Published:** 2024-11-28

**Authors:** Chengxing Wang, Zhenjuan Yin, Yu Wang, Yan Liu, Shan Zhao, Xiaoyan Dai, Ruijuan Wang, Long Su, Hao Chen, Li Zheng, Yifan Zhai

**Affiliations:** 1 Institute of Plant Protection, Shandong Academy of Agricultural Sciences, Jinan 250100, China; 2 Shandong Key Laboratory for Green Prevention and Control of Agricultural Pests, Jinan 250100, China; 3 Key Laboratory of Natural Enemies Insects, Ministry of Agriculture and Rural Affairs, Jinan 250100, China; 4 Shandong Engineering Research Center of Resource Insects, Jinan 250100, China; 5 College of Agriculture, Guizhou University, Guiyang 550025, China

**Keywords:** *Orius nagaii*, reference gene, RT-qPCR, selection, validation

## Abstract

*Orius nagaii* is a highly effective natural enemy for controlling thrips, tetranychids, aphids, and various Lepidoptera pests. Nevertheless, the molecular mechanisms underlying its interactions with host pests remain unclear. Screening for optimal reference genes is a prerequisite for using reverse transcription–quantitative polymerase chain reaction (RT-qPCR) to investigate the interrelationship. Here, ten commonly used reference genes (*Act*, *GAPDH*, *β-Tub*, *EF1-α*, *RPS10*, *RPS15*, *RPL6*, *RPL13*, *RPL32*, and *HSP90*) were selected, and their expression stability across developmental stages, tissues, temperatures, and host conditions were evaluated using *RefFinder*, which uses multiple analytical approaches (*NormFinder*, *geNorm*, the *ΔCt* method, and *BestKeeper*). The findings suggested that the most reliable normalization can be achieved by selecting the two reference genes for all conditions, with the optimal pairs being *RPS10* and *RPL32* for the developmental stage, *RPS10* and *RPS15* for tissue, *RPS10* and *RPS15* for the host, and *EF1-α* and *RPL13* for temperature. Also, the best and least stable reference genes were chosen to compare the relative transcript levels of the *TBX1* in various tissues, which exhibited considerable variation. Our findings will significantly enhance the reliability of RT-qPCR and provide a foundation for further research on the expression patterns of crucial genes that are implicated in the interaction between *O. nagaii* and its host pests.

## Simple Summary

A predatory natural enemy insect, *Orius nagaii*, has been discovered in both the northern and southern regions of China and is extensively utilized for greenhouse vegetable pest management. The precise selection of reference genes is essential for RT-qPCR analysis to elucidate the expression patterns of key genes implicated in the interaction between *O. nagaii* and its host. This study assessed the expression stability of ten candidate reference genes (CRGs) across various biological conditions and environmental stresses. Our findings revealed that the ideal reference genes were *RPS10* and *RPL32* at multiple stages of development, while *RPS10* and *RPS15* proved to be the most suitable combination for various tissues and host diets. The optimal reference gene pairing under temperature-induced stress was *EF1-α* and *RPL6*. These outcomes will offer dependable reference genes for subsequent investigations into the gene expression patterns of target genes in *O. nagaii* and closely related species.

## 1. Introduction

*Orius nagaii*, a predatory natural enemy insect, is widely distributed in both the northern and southern regions of China [1,2]. This species is known for the ability of its nymphs and adults to feed on a variety of pests, including thrips, mites, aphids, whiteflies, and other minute pests [3,4,5,6]. Additionally, *O. nagaii* has shown efficient control over the eggs and neonates of various Lepidoptera pests [7,8,9]. It plays a key role in controlling pests in greenhouse vegetables, flowers, and field cash crops, making it a natural enemy insect with high potential for development [10,11]. Semi-field cage experiments demonstrated that the efficacy of *O. nagaii* in controlling *Megalurothrips usitatus* was notably superior to that achieved through conventional chemical control measures [2], which highlighted its important value in pest control. However, the molecular mechanisms underlying the interactions between *O. nagaii* and its host pests remain unclear. Therefore, conducting gene expression analysis is of great importance in exploring its gene functions and further understanding its interaction mechanisms with host pests.

Reverse transcription–quantitative polymerase chain reaction (RT-qPCR) is a widely employed rapid detection technique of gene quantification for accurately measuring the expression levels of target genes under various biological conditions [12], which is crucial for the subsequent examination of the expression patterns of target genes in *O. nagaii*. However, when working with different experimental samples, errors may occur in the detection results of target genes due to factors such as the quality of the RNA in the starting material, the concentration of template cDNA, and experimental manipulation errors [13,14]. Hence, it is crucial to meticulously choose and validate suitable reference genes for normalization as the first step in ensuring the success of RT-qPCR analysis. Previous research has shown that many commonly used reference genes may not exhibit consistent expressions under complex experimental conditions [15]. In *Drosophila melanogaster*, the *ribosomal protein L18* (RPL18) was found to be the most stable in methanol-treated flies, while its expression was the least stable in ethyl acetate (ETOAC)-treated flies [16,17]. Meanwhile, the transcriptional level of the *glyceraldehyde-3-phosphate dehydrogenase* (*GAPDH*) gene from *Spodoptera litura* was found to be stable in different developmental periods but unstable in different tissues and geographical populations [18]. Notably, *RPL13* and *RPS18* of *Henosepilachna vigintioctomaculata* were stably expressed under various experimental conditions, including different developmental stages, tissues, hosts, and temperatures [19]. Similarly, *RPL6* was found to be stably expressed in *Mylabris sibirica* under a variety of experimental conditions [20]. However, cases of stable expression like these are relatively rare, and the change in experimental conditions may lead to fluctuations in the expression level of reference genes. Hence, it is essential to identify the best optimal reference gene with a stable expression under various research conditions for accurate target gene quantitative analysis.

Due to the lack of stability evaluation studies for reference genes in *O. nagaii*, ten frequently employed reference genes based on a previous review of 78 insect species [21], namely, β-Actin (*Act*), glyceraldehyde 3-phosphate dehydrogenase (*GAPDH*), β-tubulin (*β-Tub*), elongation factor-1-alpha (*EF1-α*), ribosomal protein S10 (*RPS10*), ribosomal protein S15 (*RPS15*), ribosomal protein L6 (*RPL6*), ribosomal protein L13 (*RPL13*), ribosomal protein L32 (*RPL32*), and heat shock protein 90 (*HSP90*) of *O. nagaii*, were chosen as candidate reference genes. *RefFinder*, which integrates four analysis methods (*NormFinder*, *geNorm*, the *ΔCt* method, and *BestKeeper*), was used to assess the expression stability of these genes under four experimental conditions (developmental period, tissue, host, and temperature). Meanwhile, the T-box transcription factor (*TBX1*) gene was used to verify the dependability of the evaluation results. This research will offer reliable reference genes for the quantitative analysis of key gene expression in the interaction between *O. nagaii* and host pests.

## 2. Materials and Methods

### 2.1. Preparation of Insects

*Orius nagaii* samples were collected from the plantation base of Shandong Academy of Agricultural Sciences (Jinan, China), classified, and identified in the Key Laboratory of Natural Enemies Insects, Ministry of Agriculture and Rural Areas, P.R. China, and were continuously propagated under the following conditions: 26 ± 1 °C, 70 ± 5% RH, and a 16/8 h (L/D) cycle [22]. *Phaseolus vulgaris* was used as the oviposition substrate for females, while nymphs and adults were fed *Sitotroga cerealella* eggs [22].

### 2.2. Sample Handling and Retrieval

Developmental stage: All phases of development were collected, including eggs, nymphs of the 1st to 5th instar, and adults. Samples were collected on the first day of each instar, with three biological replicates per sample. The quantities of individuals gathered for each repetition at various developmental phases were as follows: 90 eggs for the egg stage; 60 individuals in both the 1st and 2nd instar; 30 individuals in both the 3rd and 4th instar; and 15 individuals in both the 5th instar and adult stage.

**Figure 1:**
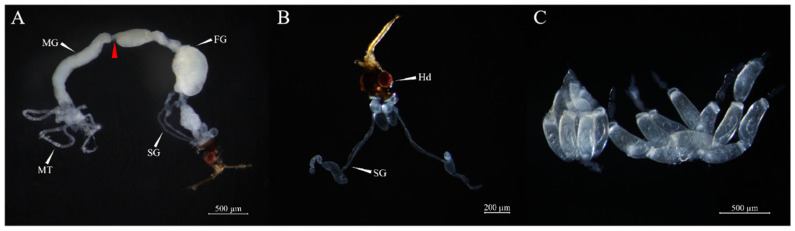
Observation and identification of various body tissues of newly emerged female adults of *O. nagaii*. (**A**) Alimentary canal. The red arrow indicates the boundary between the foregut (FG) and midgut (MG). MT, Malpighian tubule. (**B**) The salivary gland (SG) of a female adult. Hd, head. (**C**) The ovary (Ov) of a female. There are seven ovarioles in each ovary.

Tissue: One-day-old female adults were dissected under a compact stereo microscope Stemi 508 (ZEISS, Oberkochen, Germany), and different body tissues were identified and collected (Figure 1), specifically the head (Hd), salivary gland (SG), foregut (FG), midgut (MG), ovary (Ov), Malpighian tubule (MT), and residual body (RB). Three biological replicates were set per sample, with 30 insects dissected per replicate.

Host: Diet-induced stress was utilized to assess the stability of expression of candidate reference genes (CRGs) during feeding on different hosts (*S. cerealella* egg, *Corcyra cephalonica* egg, *Frankliniella occidentalis* nymph, and *Megalurothrips usitatus* nymph). Newly hatched nymphs were fed on different hosts until adult emergence, and the newly emerged adults were collected. Three biological replicates were set per sample, with 15 adults in each replicate.

Temperature: Three distinct temperatures, 8 °C, 25 °C, and 35 °C, were used for the temperature-induced stress [19]. Newly emerged adults were exposed to these temperatures for 3 h and then collected. Each sample had three biological replicates, with 15 adults per replicate.

All samples were transferred in RNase-free tubes, snap-frozen in liquid nitrogen for 2–3 min, and gathered at −80 °C for future utilization.

### 2.3. Production of cDNA Template

Sample RNA was isolated under multiple experimental conditions by following the manufacturer’s instructions for Trizol (Invitrogen, Carlsbad, CA, USA). The RNA concentration was assessed with a NanoDrop One spectrophotometer (Thermo Scientific, Waltham, MA, USA), and 1% agarose gel electrophoresis was used to check its integrity. First-strand complementary DNA (cDNA) was initially synthesized from 1 μg of sample RNA in two steps using the PrimeScript™ RT reagent Kit with the gDNA Eraser (Takara, Tokyo, Japan). The gDNA Eraser within the kit exhibits robust DNA degradation capabilities, which can effectively eliminate genomic DNA at 42 °C for 2 min. Additionally, the reverse transcription reagent includes elements that suppress the function of the DNA decomposition enzyme, so that the sample treated with gDNA Eraser can directly synthesize cDNA through the reverse transcription reaction at 37 °C, 15 min, and 85 °C, 5 s.

### 2.4. Primer Design and Verification

In our research, frequently utilized CRGs from our newly sequenced *O. nagaii* transcriptomes (unpublished data) were screened for the RT-qPCR analysis (Table 1). Utilizing conserved sequences as a foundation, precise primers were crafted using the NCBI Primer-BLAST web tool (https://www.ncbi.nlm.nih.gov/tools/primer-blast/, accessed on 24 September 2024), and the uniqueness of the primers was confirmed through 1% agarose gel electrophoresis. Amplified fragments of 80 to 200 bp of a single band were used to further assess the stability of the CRGs.

### 2.5. RT-qPCR

The RT-qPCR experiments were conducted using ChamQ Universal SYBR qPCR Master Mix (Vazyme, Nanjing, China) in a 20 µL reaction mixture. The reaction program was as follows: hold stage at 95 °C for 30 s, PCR stage (40 cycles) at 95 °C for 10 s and 60 °C for 30 s, and melt curve stage using the default system program. The melting and standard curves of the reference genes were derived using the Applied Biosystem™ QuantStudio5™ Real-Time PCR System (Thermo Scientific, Waltham, MA, USA). The amplification efficiency (E) of the RT-qPCR was calculated using the threshold cycle value (*C_t_* value) obtained from the 5-fold dilution of the template, as follows: E = (10[−1/slope] − 1) × 100% [20,23].

### 2.6. Stability Assessment

The stability of the CRGs was assessed using RefFinder (http://blooge.cn/RefFinder/, accessed on 12 October 2024) [24], which incorporates four algorithms (*NormFinder*, *geNorm*, the ΔCt method, and *BestKeeper*) to offer a thorough evaluation of the genetic stability [25,26,27,28]. The pairwise variation (Vn/n + 1) value, computed with geNorm, was employed to ascertain the ideal number of reference genes needed to normalize the target gene. A value of Vn/n + 1 < 0.15 indicates that there is no need to add another reference gene, meaning that the initial n reference genes are sufficient to achieve the standardization of the target gene.

### 2.7. Validation of Optimal Reference Genes in Various Tissues

The T-box transcription factor (*TBX1*) gene was picked as the target gene to confirm the dependability of the CRGs. Prior research has demonstrated that *TBX1* participates in the initial phase of heart development during insects’ embryonic development [29,30]. The primer sequence for the target gene is as follows: *TBX1*, F: (5′-TGGGAAGAATCACAGCATCA-3′) and R: (5′-ATCGGTATCGTTTATCGTCAAG-3′). The two most and two least stable selected reference genes were utilized to standardize the *C_t_* values of *TBX1* across various tissues. The average *TBX1* transcript level was then calculated using the 2^−∆∆Ct^ method [31]. The obtained data were statistically analyzed using one-way ANOVA in GraphPad Prism 9.0.

## 3. Results

### 3.1. Dependability of Primers for CRGs

**Figure 2:**
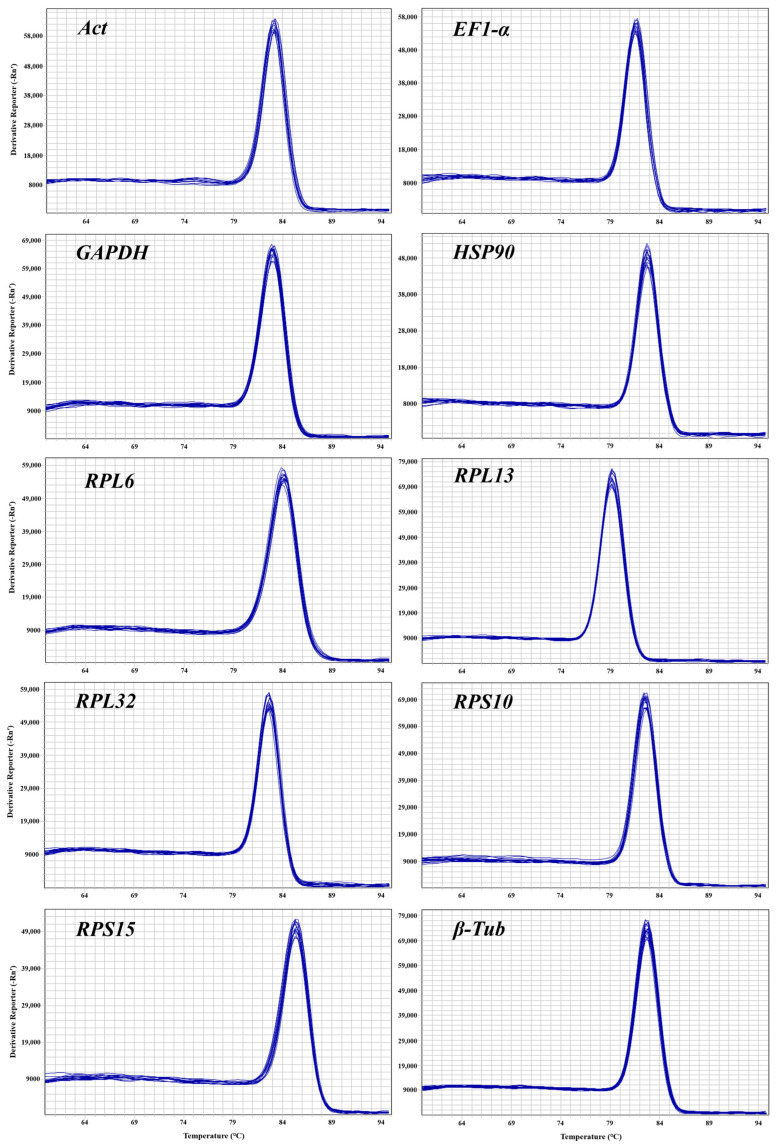
Melting profiles of the ten candidate reference genes in *O. nagaii*. A single peak indicates the absence of nonspecific amplification and primer dimerization. The *x*-axis represents the temperature, and the *y*-axis represents the fluorescence signal intensity.

Before assessing the stability of CRGs, it is essential to confirm the dependability of the primers, including the specificity and amplification efficiency. The 1% agarose gel electrophoresis of all the CRGs showed a singular band of amplified fragments (Figure S1), and the melting curves from their RT-qPCR analysis also showed a single peak (Figure 2). These results indicate that the designed primers have excellent specificity. This study provided standard curves (Figure S2) for all CRGs and calculated the primer amplification efficiency via the slope of the curve. The primer amplification efficiency ranged from 102.63 to 109.02% (Table 1), and the correlation coefficient varied between 0.9956 and 0.9999 (Table 1), indicating the high quality of the standard curve and reliable quantitative results.

### 3.2. Expression Profile of CRGs

**Figure 3:**
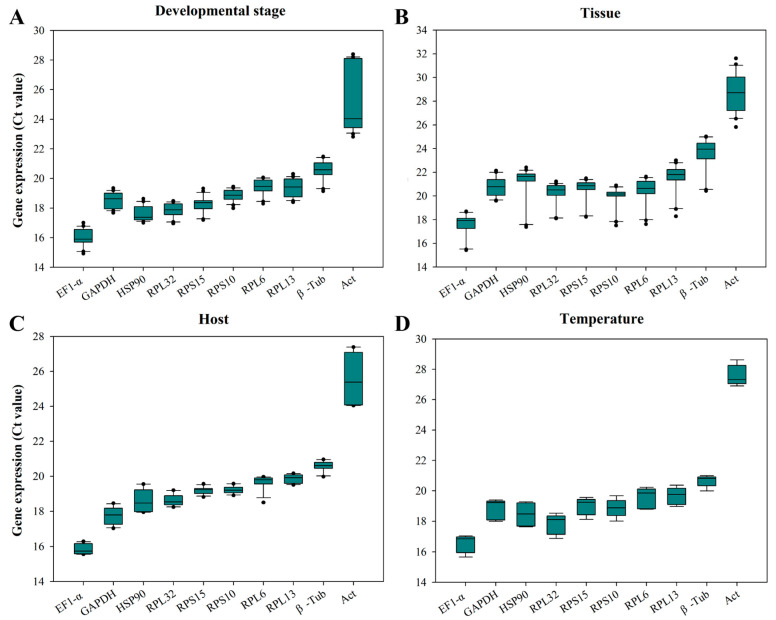
The expression profiles of candidate reference genes in *O. nagaii* under multiple experimental conditions. (**A**) Developmental stage. n = 21 for each gene. (**B**) Tissue. n = 21 for each gene. (**C**) Host. n = 12 for each gene. (**D**) Temperature. n = 9 for each gene. The *C_t_* value represents the expression level of the reference gene. The upper and lower whiskers indicate the maximum and minimum *C_t_* value, respectively. The horizontal lines inside the box represent the median. The distance between the top and bottom whisker caps of the box represents the degree of dispersion of the gene expression levels. Dots represent outliers.

The *C_t_* values of all reference genes under four experimental conditions ranged from 15.4 to 31.6. *EF1-α* was the reference gene with the highest level of richness, while *β-Tub* and *Act* were the least abundant reference genes (Figure 3). At multiple stages of development, the *C_t_* value of *RPS10* fluctuated in the smallest range, with a difference value of 1.45 (Figure 3A). In different tissues, the *C_t_* value of *GAPDH* had the smallest fluctuation range, with a difference value of 2.55 (Figure 3B). When feeding on different hosts, the *C_t_* value of *RPS10* had the smallest fluctuation range, with a difference value of 0.66 (Figure 3C). The *C_t_* value of *β-Tub* has the smallest fluctuation range under temperature-induced stress, with a difference value of 1.0 (Figure 3D). The *C_t_* value of *Act* exhibited the largest fluctuations among all experimental conditions (Figure 3).

### 3.3. Assessment of the Expression Stability of the CRGs

**Figure 4:**
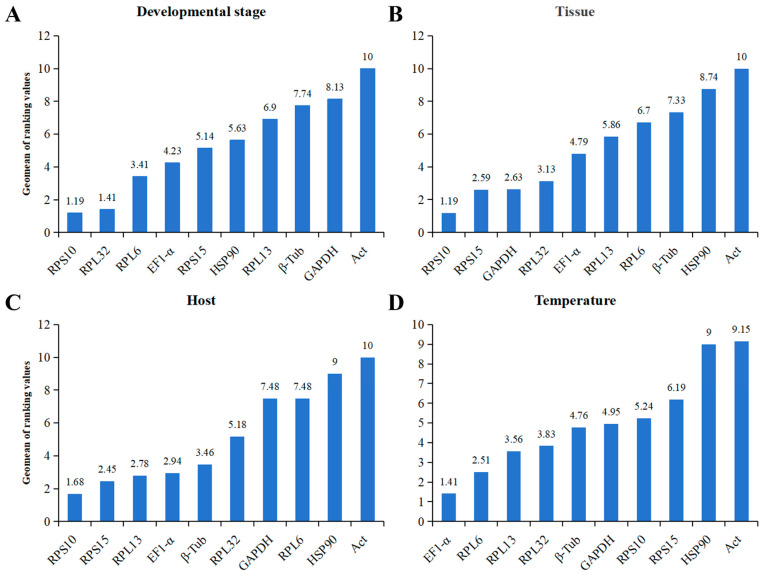
Stability of expression of ten candidate reference genes under various experimental conditions, evaluated using *RefFinder*. (**A**) Developmental stage. (**B**) Tissue. (**C**) Host. (**D**) Temperature.

Based on a comprehensive evaluation in *RefFinder*, the best to the least stable CRGs at multiple stages of development were ranked as follows: *RPS10* > *RPL32* > *RPL6* > *EF1-α* > *RPS15* > *HSP90* > *RPL13* > *β-Tub* > *GAPDH* > *Act* (Figure 4A). Within different tissues, the ranking was as follows: *RPS10* > *RPS15* > *GAPDH* > *RPL32* > *EF1-α* > *RPL13* > *RPL6* > *β-Tub* > *HSP90* > *Act* (Figure 4B). The ranking of the expression stability of the CRGs in *O. nagaii* fed with different hosts was as follows: *RPS10* > *RPS15* > *RPL13* > *EF1-α* > *β-Tub* > *RPL32* > *GAPDH* > *RPL6* > *HSP90* > *Act* (Figure 4C). Under different temperature stresses, the following ranking was determined: *EF1-α* > *RPL6* > *RPL13* > *RPL32* > *β-Tub* > *GAPDH* > *RPS10* > *RPS15* > *HSP90* > *Act* (Figure 4D). The expression stability values and rankings of all the CRGs under various experimental conditions were obtained using *Normfinder*, *geNorm*, the *ΔCt* method, and *BestKeeper* (Table 2).

### 3.4. Ideal Number of Reference Genes Across Various Experimental Conditions

**Figure 5:**
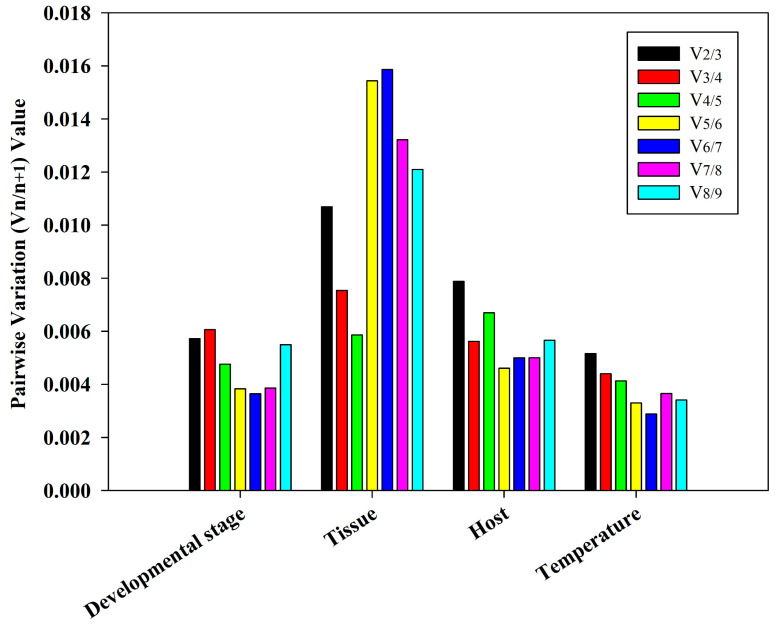
The optimal number of reference genes for accurate normalization, determined by *geNorm* analysis. A pairwise variation (Vn/n + 1) value less than 0.15 is the standard to measure the number of optimal reference genes.

The *geNorm* tool is designed to provide guidance for determining the ideal number of reference genes. A Vn/n + 1 value less than 0.15, as determined by *geNorm*, was used as our criterion for determining the ideal number of reference genes [32]. In multiple experimental conditions, the values of V_2/3_ were found to be less than 0.15, suggesting that only the two most stable reference genes are needed for normalization of the target genes (Figure 5). A correlation analysis focusing on stability (Table 2) showed that the ideal reference genes for different developmental periods were *RPS10* and *RPL32*. In different tissues, *RPS10* and *RPS15* were revealed to be the ideal reference genes. Additionally, *RPS10* and *RPS15* were the ideal housekeeping genes when feeding on different hosts. Under temperature-induced stress, *EF1-α* and *RPL6* were identified as the most suitable reference genes.

### 3.5. Confirmation of Optimal Reference Genes

**Figure 6:**
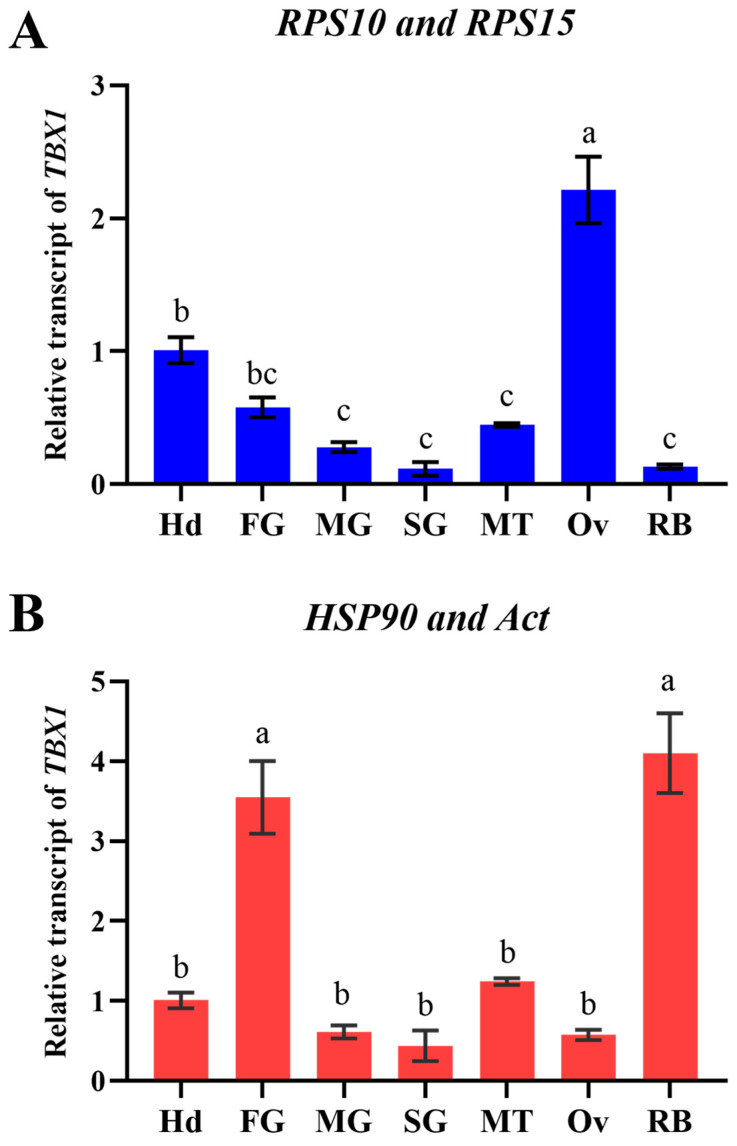
The relative transcript levels of *TBX1* in various tissues of *O. nagaii*. The transcript levels of *TBX1* were normalized using the most (**A**) and least (**B**) stable reference genes. To assess differences between tissues, a one-way ANOVA was conducted. The data are presented as mean ± SE. Different letters above the bars indicate notable variances between tissues.

To verify the dependability of the selected reference genes in different tissues, the relative expression of the *TBX1* gene was examined. It was discovered that the *C_t_* values of *TBX1* were normalized using the two most stable reference genes (*RPS10* and *RPS15*) and the two least stable reference genes (*HSP90* and *Act*), and their expression patterns in different tissues varied significantly (Figure 6). Using *RPS10* and *RPS15* as reference genes (F = 46.87; *p* < 0.0001), *TBX1* was found to be expressed at the highest level in the ovary (Ov), significantly higher than in other tissues. However, using *HSP90* and *Act* as reference genes (F = 31.50; *p* < 0.0001), the *TBX1* expression levels were higher in the foregut (FG) and residual body (RB), and significantly higher than in the Ov and other tissues.

## 4. Discussion

RT-qPCR is a widely used quantitative technique for exploring the expression patterns and relative expression levels of target genes under different biological processes and abiotic conditions [12,33]. In the future, we plan to employ RNA interference (RNAi) to investigate the roles of pivotal genes that are involved in the interaction between *O. nagaii* and host pests, and RT-qPCR will be widely used to assess the expression fluctuation of genes. The selection and verification of appropriate reference genes is crucial for ensuring reliable analysis results. However, there has been no report on the reference genes of *O. nagaii*. Prior research has found that no single gene is consistently expressed under complex experimental conditions [15]. Blindly selecting reference genes may result in difficulty detecting expression differences in target genes and could even lead to incorrect conclusions [34,35].

Here, we chose ten frequently employed reference genes and investigated their stability using various methods across four distinct experimental conditions. The results indicate that accurate normalization of target genes can be achieved by using only two of the most stable internal reference genes under various experimental conditions. *RPS10* and *RPL32* were recognized as the most stable reference genes at multiple stages of development. These genes are ribosomal protein-coding genes, involved in protein synthesis within the cell [36,37]. Our findings were consistent with previous research, where *RPL32* was found to have a stable expression at multiple developmental periods of *Bactrocera minax* [38]. However, in contrast, *RPL32* was identified to be stably expressed in *Holotrichia parallela* under different sex and photoperiodic conditions but was unstable at different developmental stages [39]. Currently, there is limited research on the stability evaluation of *RPS10*. Additionally, *RPS10* and *RPS15* were determined to be the most appropriate reference genes across various tissues and while feeding on different hosts. Previous research has also demonstrated that *RPS15* is a suitable reference gene for tissue studies of *Nilaparvata lugens* [15] and *Helicoverpa armigera* [40], as well as for studying temperature stress in *H. armigera* [41]. We found that *EF1-α* and *RPL6* were stably expressed in *O. nagaii* under temperature-induced stress, which differed from *EF1-α* being stably expressed in different tissues of *Rhodnius prolixu* [42] and *Agrilus planipennis* [43]. Similarly, *RPL6* is the most appropriate reference gene for *M.sibirica* under temperature-induced stress [20]. In summary, there is no “universal” reference gene that can be applied to various experimental conditions.

To further confirm the internal reference genes of *O. nagaii* that were screened in this study, we assessed the relative expression levels of the *TBX1* gene in various tissues. *TBX1* belongs to the T-box transcription factor gene family, which is involved in embryogenesis and organogenesis [44]. In *Andrias davidianus* and *Oncorhynchus mykiss*, *TBX1* plays a crucial role in gonadal differentiation [44,45]. Additionally, *TBX1* is involved in the initial stages of *Drosophila* embryonic development, with the strongest expression during early development [30]. Therefore, it is speculated that the *TBX1* gene may play a significant role in the reproductive process of *O. nagaii*. The findings indicated that the best appropriate reference genes (*RPS10* and *RPS15*) were used to normalize the *TBX1* gene, which had the highest expression in the ovary. This finding is consistent with our expectation based on *TBX1*’s function and is in accordance with a previous report which stated that the *TBX1* gene is highly expressed in the ovaries of *A. davidianus* larvae [45]. However, the least stable internal reference genes (*HSP90* and *Act*) were used for normalization, and the expression level of *TBX1* was found to be significantly higher in the residual body and foregut compared to other tissues, while the average expression level of *TBX1* in the ovary was only one-fourth of that achieved when using the best reference gene, which was not consistent with our expectation. Additionally, the expression pattern of *TBX1* was also found to be significantly altered in different tissues. These results highlight the importance of using appropriate reference genes to obtain reliable quantitative results for target genes. Therefore, it is necessary to screen and verify the optimal reference genes, and our study provides reliable reference genes for the quantitative analysis of functional genes that are involved in the interaction between *O. nagaii* and its host pests under strict screening conditions.

## Figures and Tables

**Table 1:** Reference genes used in this study.

Gene	Primer Sequences (5′–3′)	Length (bp)	Efficiency (%)	*R* ^2^	Linear Regression
*Act*	F: GACTGCTGAGCGTGAAATAG	107	106.73	0.9956	y = −3.1705x + 33.694
	R: GACCGTCTGGAAGTTCGTAG				
*GAPDH*	F: ATTTGTTGTTGAGCGGGATT	100	106.59	0.999	y = −3.1734x + 27.497
	R: TTGGGTTACACCGAAGACG				
*β-Tub*	F: GCGGGAAACAACTGGGCTAA	104	108.95	0.9987	y = −3.1246x + 30.147
	R: CCCTGAAGGCAATCGCAACC				
*EF1-α*	F: TGACAAAGGCTGCCGAGAA	132	103.07	0.9999	y = −3.2506x + 25.485
	R: TGGAAACACGGCTGGAGAA				
*RPS10*	F: AGAAATGCCTCCAAGCGAACT	119	107.24	0.9995	y = −3.1598x + 28.862
	R: CTACAACGAGCCCAAACACCC				
*RPS15*	F: CACTGCCGTGCTAGGAGGA	107	105.48	0.9994	y = −3.1973x + 28.448
	R: CGTTGGGCGGAGTTTCTT				
*RPL6*	F: ATGGTTGCTACGGCTGTGAA	145	109.02	0.9992	y = −3.1231x + 29.159
	R: GGAACATCTGGCTTCTGCTAT				
*RPL13*	F: ACCTTTGCCAATCCTTGTG	109	105.61	0.9982	y = −3.1944x + 28.732
	R: GGAAACCGATCCGACTTTT				
*RPL32*	F: TCTGCGAAAGGATCACCATG	148	106.00	0.9986	y = −3.186x + 27.886
	R: CCTTCGGTTTACGCCAGTTT				
*HSP90*	F: ATTCGCCGTGCTGTATCGTAA	93	102.63	0.9999	y = −3.2604x + 27.998
	R: TTCTTGGCAGCCATGTATCCC				

**Table 2:** Evaluation of expression stability of candidate reference genes under different experimental conditions.

Conditions	CRGs *	geNorm		NormFinder		BestKeeper		∆Ct		Recommendation
		Stability	Rank	Stability	Rank	Stability	Rank	Stability	Rank	
Developmental stage	*Act*	0.792	10	2.205	10	1.827	10	2.225	10	*RPS10*, *RPL32*
	*GAPDH*	0.433	9	0.443	9	0.474	6	0.811	9	
	*β-Tub*	0.341	7	0.44	8	0.515	8	0.674	8	
	*EF1-α*	0.254	4	0.29	4	0.451	5	0.59	4	
	*RPS10*	0.154	1	0.115	1	0.297	1	0.55	2	
	*RPS15*	0.285	5	0.388	7	0.421	4	0.635	5	
	*RPL6*	0.216	3	0.311	5	0.413	3	0.59	3	
	*RPL13*	0.317	6	0.362	6	0.518	9	0.649	7	
	*RPL32*	0.154	1	0.197	2	0.348	2	0.548	1	
	*HSP90*	0.366	8	0.205	3	0.493	7	0.646	6	
Tissue	*Act*	1.42	10	1.836	10	1.36	10	2.056	10	*RPS10*, *RPS15*
	*GAPDH*	0.792	6	0.548	1	0.644	2	1.236	4	
	*β-Tub*	0.515	5	1.294	8	1.02	9	1.476	8	
	*EF1-α*	0.982	7	0.763	3	0.703	5	1.279	5	
	*RPS10*	0.235	1	0.758	2	0.621	1	1.197	1	
	*RPS15*	0.235	1	0.796	5	0.654	3	1.205	3	
	*RPL6*	1.117	8	1.097	6	0.825	6	1.44	7	
	*RPL13*	0.46	4	1.165	7	0.862	7	1.373	6	
	*RPL32*	0.406	3	0.773	4	0.701	4	1.203	2	
	*HSP90*	1.261	9	1.515	9	1.007	8	1.737	9	
Host	*Act*	0.721	10	1.534	10	1.411	10	1.564	10	*RPS10*, *RPS15*
	*GAPDH*	0.455	8	0.397	7	0.385	8	0.684	7	
	*β-Tub*	0.211	1	0.388	6	0.224	4	0.61	6	
	*EF1-α*	0.364	5	0.118	1	0.245	5	0.555	3	
	*RPS10*	0.322	4	0.123	2	0.165	1	0.54	1	
	*RPS15*	0.298	3	0.143	3	0.166	2	0.548	2	
	*RPL6*	0.421	7	0.507	8	0.269	7	0.687	7	
	*RPL13*	0.211	1	0.35	5	0.223	3	0.578	4	
	*RPL32*	0.385	6	0.248	4	0.262	6	0.597	5	
	*HSP90*	0.511	9	0.748	9	0.489	9	0.849	9	
Temperature	*Act*	0.374	10	0.443	10	0.531	7	0.499	10	*EF1-α*, *RPL6*
	*GAPDH*	0.224	4	0.249	5	0.518	6	0.359	5	
	*β-Tub*	0.312	8	0.32	8	0.298	1	0.417	8	
	*EF1-α*	0.166	1	0.032	1	0.477	4	0.279	1	
	*RPS10*	0.28	7	0.255	6	0.458	3	0.365	6	
	*RPS15*	0.267	6	0.295	7	0.49	5	0.382	7	
	*RPL6*	0.166	1	0.164	2	0.545	10	0.316	2	
	*RPL13*	0.25	5	0.233	4	0.437	2	0.352	4	
	*RPL32*	0.197	3	0.223	3	0.532	8	0.342	3	
	*HSP90*	0.343	9	0.347	9	0.537	9	0.429	9	

* Candidate reference genes.

## Data Availability

The data presented in this study are available on request from the corresponding author.

## Supplementary Materials

The following supporting information can be downloaded at: https://www.mdpi.com/article/10.3390/insects15120936/s1, Figure S1: Primer specificity of candidate reference genes were determined by 1% agarose gel electrophoresis. The figure shows DNA bands of amplified fragments. M, marker; Figure S2: Standard curves for ten candidate reference genes.
