# Supplementary material for: The Selection and Validation of Reference Genes for RT-qPCR Analysis of the Predatory Natural Enemy Orius nagaii (Hemiptera: Anthocoridae)
Source: Insects. 2024 Nov 28;15(12):936. doi: 10.3390/insects15120936 (PMC11678729; doi:10.3390/insects15120936)
Supplement: Supplementary file 1 [file insects-15-00936-s001.zip › insects-3339051-supplementary.pdf]

Supplementary materials :

The Selection and Validation of Reference Genes for RT-qPCR Analysis of the Predatory Natural Enemy *Orius nagaii* (Hemiptera: Anthocoridae)

Chengxing Wang [1,2,3,4], Zhenjuan Yin [1,5], Yu Wang [1,2,3,4], Yan Liu [1,2,3,4], Shan Zhao [1,2,3,4], Xiaoyan Dai [1,2,3,4], Ruijuan Wang [1,2,3,4], Long Su [1,2,3,4], Hao Chen [1,2,3,4], Li Zheng [1,2,3,4] and Yifan Zhai [1,2,3,4,*]

[1] Institute of Plant Protection, Shandong Academy of Agricultural Sciences, Jinan 250100, China
[2] Shandong Key Laboratory for Green Prevention and Control of Agricultural Pests, Jinan 250100, China
[3] Key Laboratory of Natural Enemies Insects, Ministry of Agriculture and Rural Affairs, Jinan 250100, China
[4] Shandong Engineering Research Center of Resource Insects, Jinan 250100, China
[5] College of Agriculture, Guizhou University, Guiyang 550025, China
* Correspondence: author, E-mail: saaszyifan@163.com.

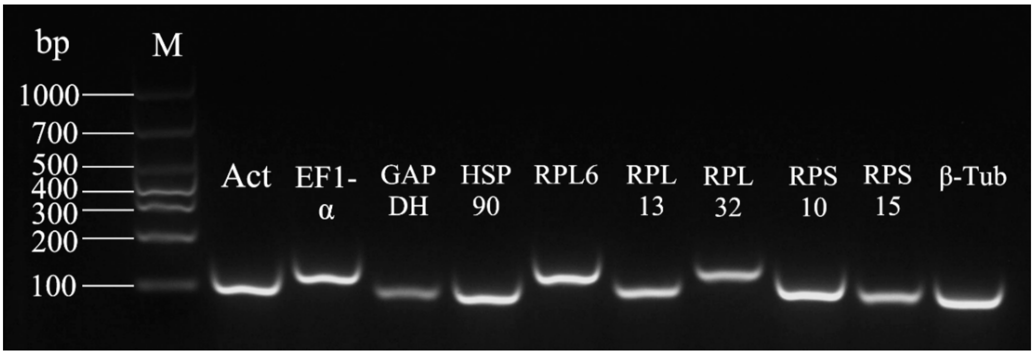

Figure S1. Primer specificity of candidate reference genes were determined by 1% agarose gel electrophoresis. The figure shows DNA bands of amplified fragments. M, marker.

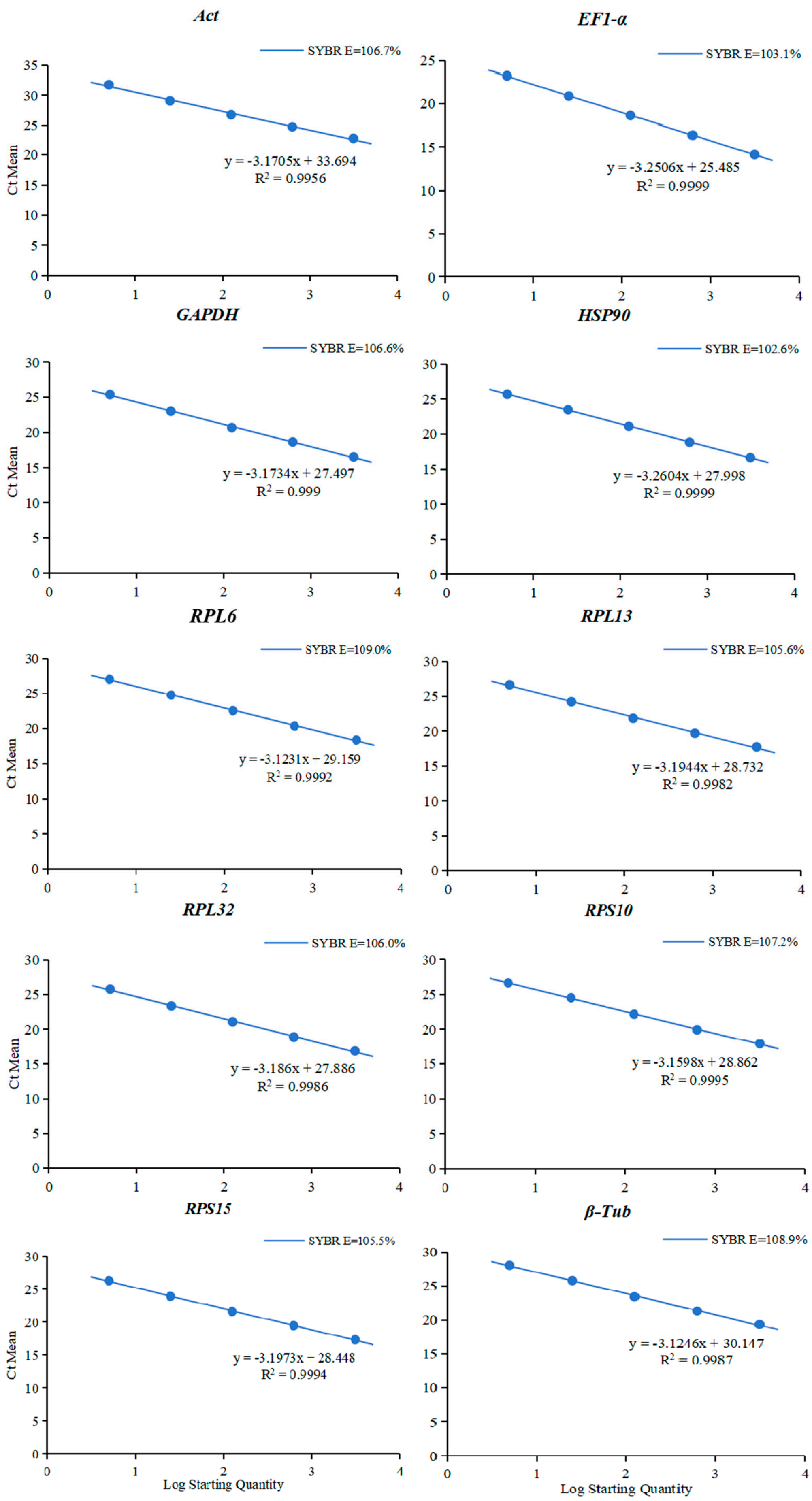

Figure S2. Standard curves for ten candidate reference genes.
